# A Machine Learning Algorithm for Quantitatively Diagnosing Oxidative Stress Risks in Healthy Adult Individuals Based on Health Space Methodology: A Proof-of-Concept Study Using Korean Cross-Sectional Cohort Data

**DOI:** 10.3390/antiox10071132

**Published:** 2021-07-16

**Authors:** Youjin Kim, Yunsoo Kim, Jiyoung Hwang, Tim J. van den Broek, Bumjo Oh, Ji Yeon Kim, Suzan Wopereis, Jildau Bouwman, Oran Kwon

**Affiliations:** 1 Department of Nutritional Science and Food Management, Ewha Womans University, 52 Ewhayeodae-gil, Seodeamun-gu, Seoul 03760, Korea; Youjin.Kim631782@tufts.edu (Y.K.); sookim726@gmail.com (Y.K.); 2 Department of Nutritional Science and Food Management, Graduate Program in System Health Science and Engineering, Ewha Womans University, 52 Ewhayeodae-gil, Seodeamun-gu, Seoul 03760, Korea; cindy.jyhwang@gmail.com; 3 Netherlands Organization for Applied Scientific Research (TNO), Department of Microbiology and Systems Biology, Utrechtseweg 48, 3704 HE Zeist, The Netherlands; tim.vandenbroek@tno.nl (T.J.v.d.B.); suzan.wopereis@tno.nl (S.W.); 4 Boramae Medical Center, Department of Family Medicine, Seoul Metropolitan Government-Seoul National University, 20 Boramae-ro 5-gil, Dongjak-gu, Seoul 07061, Korea; bo39@snu.ac.kr; 5 Department of Food Science and Technology, Seoul National University of Science and Technology, 232 Gongneung-ro, Nowon-gu, Seoul 01811, Korea; jiyeonk@seoultech.ac.kr

**Keywords:** elastic net regularized generalized linear model, diagnostic model, oxidative stress, composite biomarker

## Abstract

Oxidative stress aggravates the progression of lifestyle-related chronic diseases. However, knowledge and practices that enable quantifying oxidative stress are still lacking. Here, we performed a proof-of-concept study to predict the oxidative stress status in a healthy population using retrospective cohort data from Boramae medical center in Korea (*n* = 1328). To obtain binary performance measures, we selected healthy controls versus oxidative disease cases based on the “health space” statistical methodology. We then developed a machine learning algorithm for discrimination of oxidative stress status using least absolute shrinkage and selection operator (LASSO)/elastic net regression with 10-fold cross-validation. A proposed fine-tune model included 16 features out of the full spectrum of diverse and complex data. The predictive performance was externally evaluated by generating receiver operating characteristic curves with area under the curve of 0.949 (CI 0.925 to 0.974), sensitivity of 0.923 (CI 0.879 to 0.967), and specificity of 0.855 (CI 0.795 to 0.915). Moreover, the discrimination power was confirmed by applying the proposed diagnostic model to the full dataset consisting of subjects with various degrees of oxidative stress. The results provide a feasible approach for stratifying the oxidative stress risks in the healthy population and selecting appropriate strategies for individual subjects toward implementing data-driven precision nutrition.

## 1. Introduction

In general, aging comprises interconnected physiological processes and simultaneous unfavorable body composition and metabolic dysfunction [1]. These changes may cause persistent oxidative stress, leading to a state of chronic low-grade inflammation [2]. If not adequately controlled, oxidative stress and chronic low-grade inflammation may result in structural and functional abnormalities, leading to diet- and lifestyle-related chronic diseases, such as metabolic syndrome, cardiovascular disease, neurodegenerative disease, and certain cancers [1,2,3]. Therefore, diagnosing and identifying oxidative stress risks at an early stage is essential to optimize health and wellbeing and alleviate the increasing burden of diet- and lifestyle-related chronic diseases [4].

Many studies have identified variables, including cigarette smoking [5], aging [3], lipid peroxidation product malondialdehyde (MDA) [6,7], and body mass index (BMI) [8,9], as associated factors with oxidative stress. However, in the diagnostic setting, the response cannot be based on a single variable due to the multifactorial components found within the person [10]. According to Califf (2018), a composite biomarker may help understand the subtle changes that describe the processes and their interactions instead. Moreover, each of the multiple variables may play a critical role in the summative outcome of oxidative stress, allowing monitoring of disease progression at an early stage [11]. Researchers have used many statistical or graphical techniques like pattern recognition, principal component analysis, and partial least squares discrimination analysis to analyze and visualize multiple variables at a time [12]. However, these techniques often define the axis based on the variation or line that best separates the defined groups, thus having no biological meaning [13]. Bouwman et al. (2012) recently proposed a statistical visualization method, named ‘health space’, that addresses this issue by projecting individual subjects’ health status based on predefined biological processes determined by multivariate parameterization.

The rapid development in big data analysis and artificial intelligence has recently begun to unlock clinically relevant information from cohort data, journals, and clinical practices hidden in a large volume of these, assisting clinical practice by providing up-to-date medical information or reducing diagnostic and therapeutic errors [14,15,16]. However, to the best of our knowledge, no studies have applied machine learning (ML) algorithms to serve as a starting point to reduce the risk of oxidative stress-related chronic diseases in advance and answer concerning data-driven precision nutrition. We tackle this problem by exploring a proof-of-concept study to test the utilization of the health space methodology for extensive population studies. Furthermore, we used ML approaches to develop a model that discriminates oxidative stress risks with strong prediction power and interpretability by selecting valuable features from the full spectrum of diverse and complex data. Subsequently, we validated the proposed diagnostic model in a separate hold-out test set of subjects. This study may initiate and facilitate evidence-based data-driven precision nutrition.

## 2. Materials and Methods

### 2.1. Study Population

This study used a retrospective cohort design to analyze 2454 subjects, who first came to Seoul Metropolitan Government–Seoul National University Boramae Medical Center (Seoul, Republic of Korea) for regular general health check-ups between 1 April 2015 and 31 August 2018. A total of 1328 subjects were included after excluding (1) subjects with missing data on any independent variables selected for analysis (*n* = 618), (2) subjects aged under 20 years old (*n* = 5), (3) same persons visited twice (*n* = 117), and (4) patients diagnosed with diseases excluding oxidative diseases (*n* = 386). The Institutional Review Boards of Seoul National University Boramae Medical Center (approval number 20140929/26-2014-118/102) and Ewha Womans University (approval number 86-8) approved this study. We received written informed consent from all subjects and performed all procedures following the relevant guidelines and regulations.

### 2.2. Data Collection

We extracted a total of 43 features from the record and developed a structured database. Data on general characteristics included age, sex, smoking status, alcohol consumption, and all diagnosed diseases. The overall diet quality and physical activity were determined by the recommended food score (RFS) [17] and validated Korean version of the International Physical Activity Questionnaire [18], respectively. Anthropometric data included body fat percentage and BMI. Biochemical analyses of serum samples included albumin, alkaline phosphatase (ALP), blood bilirubin, blood urea nitrogen (BUN), creatinine, C-reactive protein (CRP), γ-glutamyl transferase (GGT), glutamate oxaloacetate transaminase (GOT), glutamate pyruvate transaminase (GPT), glycosylated hemoglobin (HbA1c), low-density lipoprotein cholesterol (LDL-C), total protein, total cholesterol (TC), and uric acid (UA). Complete blood count (CBC) data included basophils, eosinophils, erythrocyte sedimentation rate (ESR), hematocrit (Hct), hemoglobin (Hb), lymphocytes, mean corpuscular hemoglobin (MCH), mean corpuscular hemoglobin concentration (MCHC), mean corpuscular volume (MCV), monocytes, neutrophils, platelet count, platelet distribution width (PDW), red blood cell count (RBC), red blood cell distribution width (RDW), and white blood cell count (WBC).

### 2.3. MDA Analysis

We analyzed the MDA levels in plasma, erythrocyte, and urine using an HPLC system (Shiseido, Tokyo, Japan) equipped with a fluorescence detector (emission = 527 nm, excitation = 551 nm). A Capcell Pak C18 UG120 column (4.6 mm × 250 mm, 5 μm particle size; Shiseido) was used for separation under isocratic elution with 50 mM phosphate buffer [19,20,21,22].

### 2.4. Development and Validation of ML Algorithm

**Figure 1:**
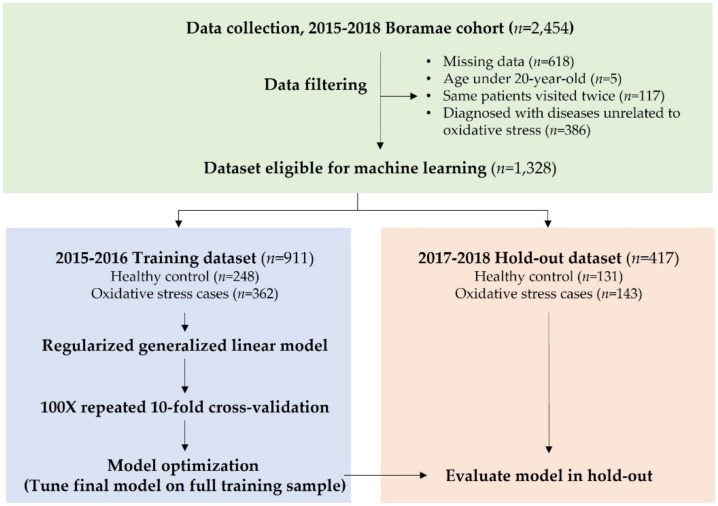
Schematic of the analysis pipeline. The eligible datasets were split into training and hold-out validation datasets. The least absolute shrinkage and selection operator (LASSO)/elastic net regression algorithm was trained using the training dataset and further evaluated in the hold-out validation dataset.

A schematic of the analysis pipeline is presented in Figure 1. We first split the eligible dataset (*n* = 1328) into training (*n* = 911) and hold-out (*n* = 417) datasets after removing the duplicate samples. The training dataset consisted of subjects who received a regular general health check-up at the Seoul Metropolitan Government–Seoul National University Boramae Medical Center in 2015–2016. The most recent dataset of the 2017–2018 period was used as the hold-out test set. Notably, the separate and independent hold-out dataset was used only for validating the final model to ensure no bias in the model development.

To obtain binary performance measures, we formed two reference groups in the training and hold-out datasets by adopting a health space model [13]. The healthy controls were defined as subjects having neither metabolic syndrome nor its components nor physician-diagnosed medical conditions. Extensive phenotyping of oxidative stress-related diseases was made based on the previously published data. As a result, the oxidative disease cases were defined as subjects with metabolic syndrome [1], dyslipidemia [9], hypertension [9], intermediate coronary syndrome [5], stroke [7], diabetes mellitus [23], or liver cirrhosis [24], among all subjects diagnosed as having diseases (Supplementary Materials Figure S1). We used the disease terminology presented by the Human Phenotype Ontology [25].

Considering the high-dimensional and collinear characteristics of the data, we employed the regularized generalized linear model (GLM) with the least absolute shrinkage or selection operator (LASSO) and elastic net penalties using the R package “glmnet” [26]. The mixing parameter alpha was set to 0.5, reflecting the balance between the ridge and LASSO penalties of the two approaches [27]. The resulting multivariate regression model was validated by 10-fold stratified cross-validation (CV) to avoid overfitting [28]. This procedure was repeated 100 times to improve stability by increasing the number of evaluations from 10 to 1000 [29]. Then, based on the one-standard-error rule, we calculated the optimal value of lambda (λ) and the minimum misclassification rate (lambda.1se) to tune the elastic net and LASSO regressions [30,31]. The best-performing model was identified by comparing the two best candidates derived from each elastic net and LASSO regression using the area under the receiver operating characteristic curve (AUC). Finally, external validation of the best performing model was carried out by calculating AUC, specificity, sensitivity, accuracy, negative predictive value (NPV), positive predictive value (PPV), positive clinical utility index (CUI+), and negative clinical utility index (CUI-) [32,33].

Furthermore, we investigated calibration to confirm whether the predicted probabilities agree with the observed probabilities [34]. To this end, the final prediction algorithm was applied to the entire dataset (*n* = 1328), stratified into four categories according to the number of metabolic syndrome risk factors (0, 1, and 2) and the presence of oxidative stress diseases. Next, we applied our final prediction algorithm to the subjects excluded from this study due to other conditions unrelated to oxidative stress. Then, Duncan’s post hoc test was used to delineate group differences further.

### 2.5. Statistics

All statistical analyses were performed using the R software (version. 3.6.1; R Foundation for Statistical Computing) [35]. The results were expressed descriptively as means and standard deviation (SD) for continuous variables and number (percentage) for categorical variables. Differences between healthy controls and oxidative disease cases were compared by the Student’s *t*-test for continuous variables and Chi-square test for categorical variables. Statistical significance was set at *p* < 0.05.

## 3. Results

### 3.1. Characteristics of the Reference Groups

Out of 1328 subjects, 884 samples were extracted as healthy controls (*n* = 379, 28.5%) and oxidative disease cases (*n* = 505, 38%) to develop and validate the ML algorithm discriminating oxidative stress risks in the healthy population. Table 1 compares the 43 features derived from these two reference groups in the training and hold-out dataset, respectively. The results indicate the separable features of the groups. In both datasets, the subjects in the oxidative disease cases were older, with a higher percentage of male subjects and smokers than the healthy controls. The oxidative disease cases had significantly higher BMI and body fat percentages than the healthy controls. This trend was similar for the ALP, BUN, creatinine, CRP, GGT, GPT, HbA1c, and UA levels. In contrast, albumin, bilirubin, LDL-C, TC, and total protein levels did not differ between the two groups. For CBC data, Hb, Hct, MCHC, RBC, and WBC were significantly higher in the oxidative disease cases than in the healthy controls.

### 3.2. Developing an ML Algorithm for Discriminating Oxidative Stress Risks in the Healthy Population

**Figure 2:**
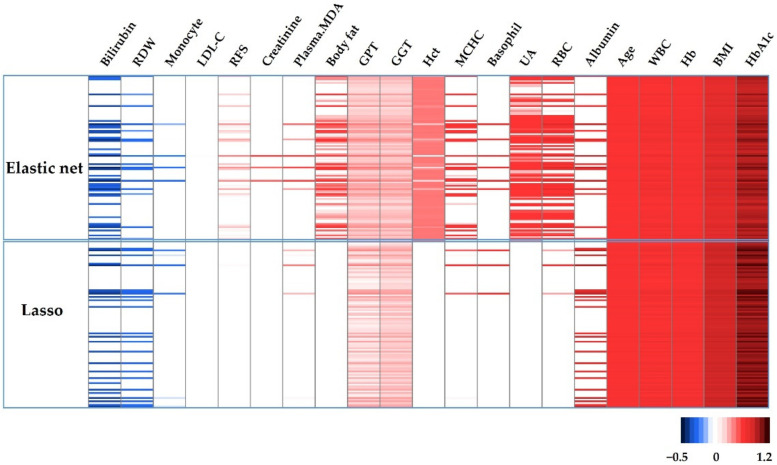
Regression coefficients for the features selected in 200 different 10-CV least absolute shrinkage and selection operator (LASSO)/elastic net regularized generalized linear models. The color scale beneath the heatmap represents a range of coefficient values from negative (blue) to red (positive). CV, cross-validation; RDW, red blood cell distribution width; LDL-C, low-density lipoprotein cholesterol; RFS, recommended food score; MDA, malondialdehyde; GPT, glutamate pyruvate transaminase; GGT, γ-glutamyl transferase; Hct, hematocrit; MCHC, mean corpuscular hemoglobin concentration; UA, uric acid; RBC, red blood cell count; WBC, white blood cell count; Hb, hemoglobin; BMI, body mass index; HbA1c, glycosylated hemoglobin.

The heatmap presented in Figure 2 shows all features and corresponding coefficients obtained from the training dataset using logistic regression with elastic net (upper) and LASSO (lower) penalties. Ten-fold CV was run 100 times for each penalty. The color scale beneath the heatmap represents a range of coefficient values, where blue is negative and red is positive. Twenty-one out of a total of 43 features were extracted at least once within the 200 individual replications. Age, BMI, GGT, GPT, Hb, HbA1c, and WBC were the most consistently extracted features across all 200 models.

**Figure 3:**
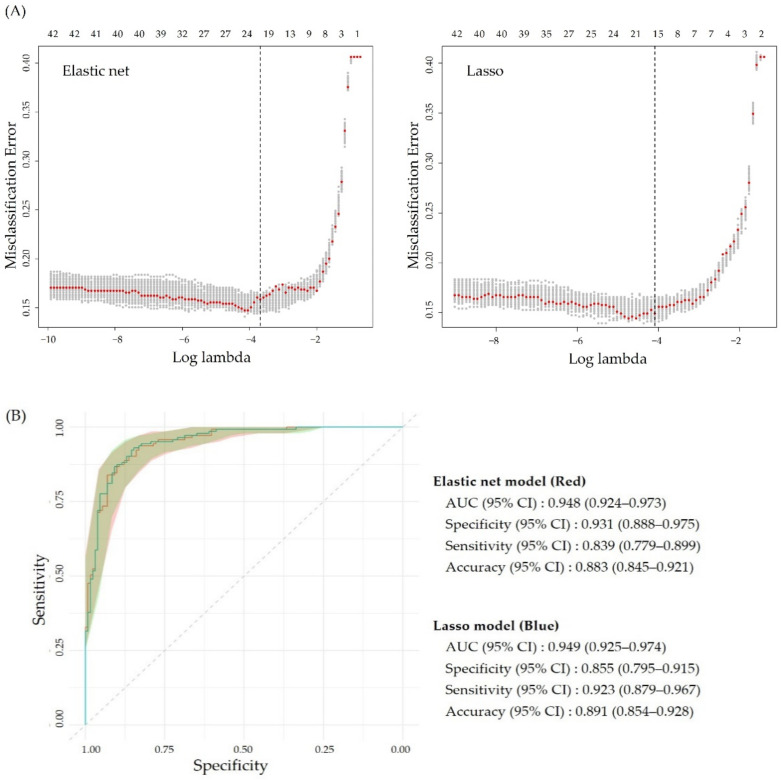
Selection of the best performing model. (**A**) Cross-validation estimate of the mean squared prediction error for elastic net (left) and least absolute shrinkage and selection operator (LASSO, right), as a function of log λ. Grey bars are one-standard errors, and the vertical dashed lines are the location of the minimum misclassification error. Numbers on the top of the panel indicate the number of features selected. (**B**) Receiver operating characteristic curve for the elastic net and LASSO model on hold-out samples. The diagonal line represents the reference line of 0.5. AUC, area under the curve; CI, confidence interval.

The CV results are presented in Figure 3A, depicting the mean squared prediction error against log λ with one-standard error bars. The vertical dashed lines indicate the location of the minimum misclassification error (lambda.1se) selected by a “one-standard-error” rule. The elastic net regression gives lambda.1se = 0.0253, and the LASSO regression yields lambda.1se = 0.0167, indicating that LASSO produced a more regularized model compared with the elastic net model. Figure 3B compares the performance of the elastic net and LASSO models using AUC, validating the above findings that the LASSO model (AUC 0.949, 95% confidence interval [CI] 0.925–0.974) performed slightly better than the elastic net model (AUC 0.948, 95% CI 0.924–0.973). The best LASSO regression model contains 16 features (age, plasma MDA, BMI, RFS, HbA1c, GPT, GGT, bilirubin, albumin, WBC, RBC, Hb, RDW, monocytes, basophils, and MCHC). The feature with the highest negative coefficient value was bilirubin, while those with the highest positive coefficient value were HbA1c and albumin. In contrast, the best elastic net regression model contains four more features, including creatinine, UA, body fat percentage, and LDL-C.

### 3.3. Internal and External Validation of the Best Performing Model

**Figure 4:**
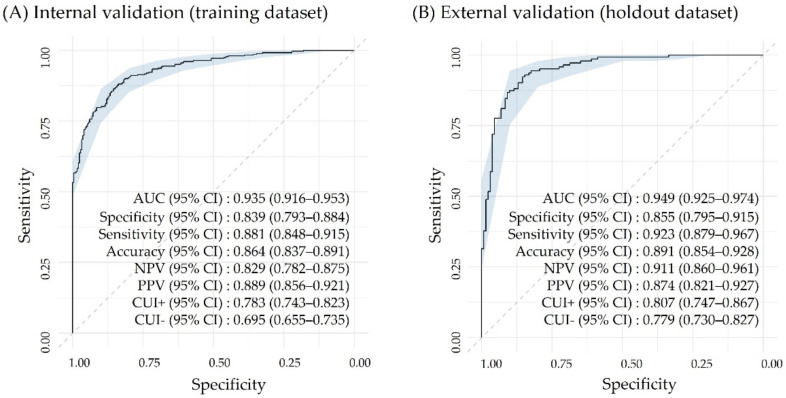
Performance of the best performing machine learning model on two datasets. (**A**) Training dataset and (**B**) hold-out dataset. The diagonal line represents the reference line of 0.5. Light blue indicates 95% CIs of the ROC curve estimate. CI, confidence interval; ROC, receiver operating characteristic.

Figure 4A shows the diagnostic performance of our final best performing model in the training dataset (*n* = 610), which consisted of 248 healthy controls and 362 oxidative disease cases. The result was excellent, presenting an AUC of 0.935 (95% CI, 0.916–0.953), specificity of 0.839 (95% CI, 0.793–0.884), sensitivity of 0.881 (95% CI, 0.848–0.915), accuracy of 0.864 (95% CI, 0.837–0.891), NPV of 0.829 (95% CI, 0.782–0.875), PPV of 0.889 (95% CI, 0.856–0.921), CUI+ of 0.783 (95% CI, 0.743–0.823), and CUI- of 0.695 (95% CI, 0.655–0.735). Figure 4B demonstrates the outstanding discrimination performance of our final model validated using the hold-out dataset (*n* = 274), which comprised 131 healthy controls and 143 oxidative disease cases. The result was even better, with an AUC of 0.949 (95% CI, 0.925–0.974), specificity of 0.855 (95% CI, 0.795–0.915), sensitivity of 0.923 (95% CI, 0.879–0.967), accuracy of 0.891 (95% CI, 0.854–0.928), NPV of 0.911 (95% CI, 0.86–0.961), PPV of 0.874 (95% CI, 0.821–0.927), CUI+ of 0.807 (95% CI, 0.747–0.867), and CUI- of 0.779 (95% CI, 0.73–0.827). Statistically, the healthy controls in the training set and those in the hold-out set had compatible variables, except for age, albumin, BUN, HbA1c, total protein, Hct, MCHC, RBC, and erythrocyte MDA. However, when we compare those values with the reference guideline level, they were within the normal ranges (Table S1).

### 3.4. Application of the Best Performing Model to All Subjects in the Whole Dataset for Testing Discrimination Power

**Figure 5:**
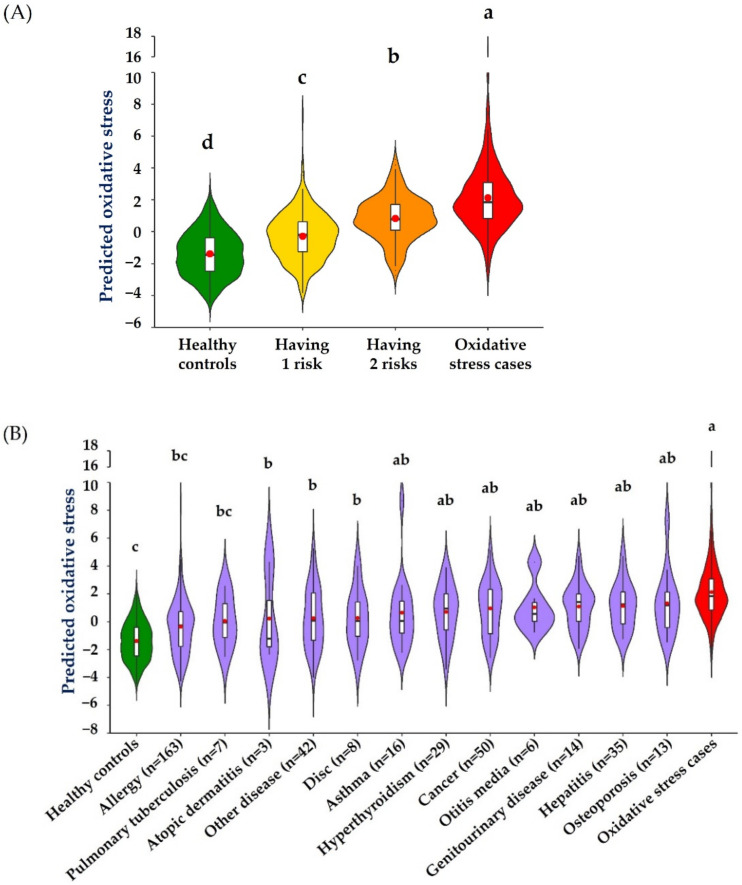
Application of the final model for qualitative diagnosis of oxidative stress risks. (**A**) Calibration of oxidative stress risks of subjects having zero (*n* = 379), one (*n* = 282), and two metabolic risk factors (*n* = 162) and oxidative disease cases (*n* = 505). (**B**) Application of oxidative stress risks of subjects with other diseases not related to oxidative stress (*n* = 386) in comparison to the healthy controls (*n* = 379) and oxidative disease cases (*n* = 505). The violin plot represents the distribution of data by using the kernel density function, and the width of the violin plot represents the sample size at this level. The red dot on each box plot represents the mean value. The box in the violin plot represents the median and quartile; the extension from the thin black line represents the 95% confidence interval. The different lowercase letters indicate a significant difference between the health status by Duncan’s test (*p* < 0.05).

Figure 5A is a violin plot illustrating the diagnostic values for oxidative stress of all individuals stratified by four categories based on the number of metabolic syndrome risk factors and the presence of oxidative stress diseases. The results confirmed that our final model can suitably define healthy and oxidative disease categories, which seems to be better than the traditional separation based on clinical diagnosis or the metabolic syndrome definition. Moreover, our final model can identify individuals with higher metabolic risks as having higher oxidative stress risks toward the oxidative disease cases (*p* for trend < 0.001). The result presented in Figure 5B shows the diagnostic values for oxidative stress of the subjects excluded from this study due to the presence of other diseases unrelated to oxidative stress. The other disease groups colored in purple were significantly different or at least tended to differ from either the healthy controls or the oxidative stress cases, but it was challenging to calibrate the apparent magnitude of oxidative stress risks.

## 4. Discussion

Given the need to better discriminate high-risk individuals for diet- and lifestyle-related chronic diseases in the general population, we developed and validated an ML model to diagnose individuals’ oxidative stress risks at an early stage. Many prediction models for disease diagnosis or prognosis have emerged [10,36], but they do not enable individuals to monitor disease deterioration. The present study was initiated as proof of the health space model, a statistical model for analyzing and visualizing the treatment effects of functional foods in individual subjects based on predefined biological processes [13]. The health space model was applied to depict subjects’ oxidative stress status by evaluating the summative outcomes of biological processes, thus avoiding erroneous conclusions [11]. Furthermore, it was expanded to derive binary samples of healthy controls and oxidative disease cases from the Boramae cohort data.

As oxidative stress is complex and multifactorial, a composite biomarker might facilitate diagnosing and risk-stratifying subjects with high oxidative stress levels than any single biomarker [6,37,38,39,40,41]. The current study used ML models in the multivariate statistical analysis rather than the traditional statistical approaches. The advantages of ML models over traditional statistical approaches include their ability to consider interactions between features and explore combinations that might not be apparent [42]. Among many algorithms, the GLM-based technique has mainly been used for the text mining of electronic health reports and developing a prediction model in health care, providing a simple and interpretable description [43]. However, the performance of GLMs has been indicated as unsatisfactory because they are prone to overfitting and sensitive to outliers [44]. Alternatively, a graph learning-matching network (GLMNet) that fits a GLM via penalized maximum likelihood can be used to overcome the limitations of naïve GLMs [28,45,46]. In this current study, we applied the two representative regularization methods called LASSO and elastic net. Our results showed that the LASSO regression better predicted oxidative stress risks than the elastic net regression.

In a data mining algorithm, proper internal validation is essential to prevent model overfitting and reduce potential false-positive findings [47]. The split-sample approach is a straightforward and popular method in which the training data are randomly split into two parts for developing a model and measuring its performance. The CV is a more sophisticated approach with the advantage of intuitively being regarded as an extension of the split-sample method [48]. In the current study, we applied CV for internal validation with a 10% fraction to test a model developed on 90% of the sample in this context. This procedure was repeated 100 times in the training dataset to improve CV stability. Such stratification is ideal for keeping the test dataset intact and bringing it out only at the end of the data analysis. However, because the test dataset was used repeatedly in this study, we determined the regularization parameters to choose the most parsimonious model whose error is no more than one standard error [31,48]. As a result, we obtained a final transparent and easy-to-interpret diagnostic model composed of a combination of 16 features representing the composite of anthropometrical, biochemical, and clinical data.

We note that we reserved a separate and independent hold-out dataset for external validation of the final model. AUC analysis was performed to assess the final model performance, presenting an AUC value of 0.949 in the validation dataset versus 0.935 in the training dataset. In the context of discrimination, AUC values of 1.0, 0.9–0.99, 0.8–0.89, 0.7–0.79, 0.51–0.69, and 0.5 can be interpreted as perfect, excellent, good, fair, poor, and of no value, respectively [49]. Therefore, our model may provide appropriate selection criteria for individuals to implement data-driven precision nutrition. Contrary to our result, some studies that explored a global oxidative stress index (Oxidative-INDEX) did not reach beyond the good prediction level. Park et al. [50] created an oxidative stress score consisting of MDA, oxidized low-density lipoprotein, and 8-epi-prostaglandin F2α. The AUC value was 0.75 when combined with single nucleotide polymorphisms to predict obesity in the Korean population. In another study, the Oxidative-INDEX was calculated in patients with coronary artery disease by measuring overall pro- and antioxidant exposure balance after *z*-score standardization [51]. It showed significant associations of the score with diabetes, smoking habit, hypercholesterolemia, aging, and CRP at the multivariate regression analysis, suggesting the potential use of the Oxidative-INDEX in preventing, diagnosing, and treating coronary artery disease. However, validation remained undone, and further investigation was needed in the general population.

There is increasing interest in the application of ML for nutritional science to prevent and improve diet- and lifestyle-related chronic diseases. However, to our knowledge, no prior published studies included the application of ML algorithms for quantifying health conditions and providing appropriate strategies to individuals for implementing data-driven precision nutrition in the context of our study. Our model was able to quantitatively stratify oxidative stress burden, as expected. It could even distinguish healthy individuals from most individuals with diseases unrelated to oxidative stress, although to a relatively lesser degree of preciseness. However, this study had several limitations. First, even though the model performance was excellent, as indicated by CV discrimination and external validation, there was partial overlap between the groups categorized by the number of metabolic syndrome risk factors (0, 1, and 2) and the presence of oxidative stress diseases. Other statistical considerations may have to be enjoined to solve this problem. Second, this study was conducted using the dataset obtained from adults residing in the Republic of Korea. Thus, the results of this study may not be generalizable to all ethnic populations. Further investigations are underway to develop and validate an oxidative stress predictor in a more extensive and diverse multi-ethnic population using the same ML techniques. Third, in this study, we excluded those subjects with missing observations rather than replacing missing values with imputation methods. This approach may induce the problem of reducing statistical power or yielding biased estimates if in traditional epidemiology and clinical research [52]. Therefore, we performed multiple imputation using the partially observed cases by chained equations in R package mice [53]. Although the number of selected features increased from 16 to 22 after imputation, most of them were duplicated with the model presented here. In addition, the diagnostic performance was compatible with each other (Figure S2). Last, the primary aim of creating the composite predictor was to facilitate identifying an individual’s health condition and predicting longitudinal outcomes of an individual’s health risks. However, by using a cross-sectional dataset, our model allowed us to develop only a diagnostic model for distinguishing oxidative stress levels of each individual. We may need a population-based longitudinal cohort dataset that follows individuals and tracks the progression of chronic diseases related to oxidative stress in order to develop a prognostic risk model and use it as a practical reference for subsequent personalized health decisions. The prognostic model is likely to be conveyable to the general public if appropriately validated.

## 5. Conclusions

The proposed ML algorithm is based on the cross-sectional data obtained from the Boramae medical center cohort in Korea. It allowed the development of a composite diagnostic model for oxidative stress at the individual level. The resulting predictor comprised 16 features and was proved to have excellent performance in quantifying oxidative stress risks. Considering the importance of these findings in the context of precision nutrition, the limitations of this study create opportunities for further research to enhance and expand our current understandings. The present study is the first to report a feasible approach for stratifying oxidative stress risks in a healthy population, providing appropriate strategies to engage in proactive health management to prevent diet- and lifestyle-related chronic diseases.

## 6. Patents

The application numbers of the patent related to this work are 10-2020-0071809 and PCT/KR2021/007312.

## Figures and Tables

**Table 1:** Comparison between healthy controls and oxidative disease cases included in the training and hold-out datasets for the machine learning algorithm discriminating oxidative stress risks in the healthy population.

Features	2015–2016 Training Set (*n* = 610)		*p*-Value	2017–2018 Hold-Out Set (*n* = 274)		*p*-Value
	Healthy Controls (*n* = 248)	Oxidative Disease Cases (*n* = 362)		Healthy Controls (*n* = 131)	Oxidative Disease Cases (*n* = 143)	
**General characteristics (10)**						
Age (years)	42.7 ± 10.4	52.5 ± 10.2	<0.0001	38.3 ± 10.0	52.4 ± 10.1	<0.0001
Sex (males; *n*, %)	101 (40.7)	264 (72.9)	<0.0001	47 (35.9)	102 (71.3)	<0.0001
Current smoker (*n*, %)	23 (9.3)	97 (26.8)	<0.0001	5 (3.8)	15 (10.5)	0.034
Smoking duration (y)	4.5 ± 9.0	11.7 ± 13.1	<0.0001	3.1 ± 6.8	9.3 ± 12.6	<0.0001
Smoking pack-year	3.3 ± 7.6	9.5 ± 12.7	<0.0001	2.4 ± 6.4	9.3 ± 15.7	<0.0001
Current drinker (*n*, %)	145 (58.5)	224 (61.9)	0.397	78 (59.5)	82 (57.3)	0.712
RFS	21.6 ± 8.3	24.7 ± 8.7	<0.0001	20.5 ± 8.7	22.5 ± 9.0	0.069
Physical activity (m/d)	128.5 ± 120.1	133.9 ± 118.9	0.581	119.2 ± 113.8	81.0 ± 107.0	0.005
BMI (kg/m^2^)	21.5 ± 2.2	25.6 ± 4.1	<0.0001	21.2 ± 2.2	25.4 ± 3.7	<0.0001
Body fat (%)	25.5 (6.4)	28.6 (6.9)	<0.0001	26.0 (6.4)	29.3 (6.5)	<0.0001
**Biochemical characteristics (14)**						
Albumin (g/dL)	4.3 ± 0.2	4.4 ± 0.2	0.089	4.4 ± 0.2	4.5 ± 0.2	0.079
AP (IU/L)	62.5 ± 17.8	72.6 ± 18.5	<0.0001	62.3 ± 17.2	70.1 ± 17.8	<0.0001
Bilirubin (mg/dL)	1.2 ± 0.5	1.1 ± 0.4	0.129	1.1 ± 0.4	1.1 ± 0.4	0.237
BUN (mg/dL)	12.8 ± 3.5	13.9 ± 3.4	<0.0001	11.6 ± 2.8	13.8 ± 3.3	<0.0001
Creatinine (µmol/L)	68.2 ± 14.1	77.0 ± 15.5	<0.0001	66.1 ± 12.6	77.6 ± 15.8	<0.0001
CRP (mg/dL)	0.1 ± 0.2	0.2 ± 0.3	<0.0001	0.1 ± 0.3	0.2 ± 0.4	<0.0001
GT (IU/L)	18.4 ± 15.0	38.3 ± 38.2	<0.0001	17.7 ± 13.7	39.7 ± 36.0	<0.0001
GOT (IU/L)	22.6 ± 6.8	30.5 ± 18.4	<0.0001	26.7 ± 56.0	31.2 ± 17.0	0.372
GPT (IU/L)	18.9 ± 12.0	35.5 ± 32.4	<0.0001	18.6 ± 12.3	34.2 ± 24.2	<0.0001
Glycosylated Hb (%)	5.4 ± 0.3	6.0 ± 0.9	<0.0001	5.3 ± 0.3	5.9 ± 0.9	<0.0001
LDL-C (mmol/L)	3.0 ± 0.7	3.1 ± 0.9	0.223	3.0 ± 0.7	3.2 ± 1.1	0.028
TC (mmol/L)	5.0 ± 0.8	5.1 ± 1.1	0.293	5.0 ± 0.8	5.2 ± 1.1	0.089
Total protein (g/dL)	7.1 ± 0.4	7.2 ± 0.4	0.283	7.2 ± 0.3	7.3 ± 0.4	0.081
Uric acid (mg/dL)	4.8 ± 1.2	5.6 ± 1.4	<0.0001	4.7 ± 1.1	5.6 ± 1.3	<0.0001
**Complete blood count data (16)**						
Basophils (%)	0.4 ± 0.3	0.4 ± 0.3	0.504	0.5 ± 0.3	0.4 ± 0.3	0.273
Eosinophils (%)	2.6 ± 2.2	2.8 ± 2.2	0.273	2.7 ± 2.0	2.9 ± 2.5	0.419
ESR (mm/h)	9.0 ± 8.2	9.6 ± 8.4	0.380	9.4 ± 11.9	11.8 ± 11.1	0.090
Hematocrit (%)	41.4 ± 4.2	44.2 ± 3.8	<0.0001	42.7 ± 3.7	44.9 ± 3.7	<0.0001
Hemoglobin (g/dL)	13.9 ± 1.6	15.0 ± 1.5	<0.0001	14.2 ± 1.5	15.1 ± 1.4	<0.0001
Lymphocytes (%)	35.3 ± 8.8	35.4 ± 8.1	0.883	36.4 ± 7.9	36.3 ± 7.6	0.893
MCH (pg)	30.3 ± 1.9	30.8 ± 1.5	<0.0001	30.0 ± 1.9	30.4 ± 1.3	0.094
MCHC (g/dL)	33.5 ± 1.0	34.0 ± 0.9	<0.0001	33.2 ± 0.9	33.5 ± 0.9	0.002
MCV (fL)	90.4 ± 4.4	90.8 ± 3.9	0.209	90.6 ± 4.7	90.7 ± 3.4	0.830
Monocytes (%)	5.7 ± 1.5	5.6 ± 1.4	0.424	5.8 ± 1.4	5.9 ± 1.5	0.709
Neutrophils (%)	56.0 ± 9.7	55.8 ± 8.6	0.775	54.7 ± 8.4	54.6 ± 8.5	0.905
Platelets (×10^3^/µL)	249.1 ± 51.9	247.9 ± 53.4	0.781	258.5 ± 54.0	258.4 ± 86.8	0.995
PDW (%)	11.8 ± 1.6	11.9 ± 1.5	0.412	11.8 ± 1.5	12.2 ± 1.6	0.016
RBC count (×10^6^/µL)	4.6 ± 0.4	4.9 ± 0.5	<0.0001	4.7 ± 0.4	5.0 ± 0.4	<0.0001
RDW (%)	13.1 ± 1.0	12.9 ± 0.6	0.014	13.1 ± 1.1	12.9 ± 0.6	0.188
WBC count (×10^3^/µL)	5.2 ± 1.3	6.2 ± 1.7	<0.0001	5.2 ± 1.3	6.1 ± 1.8	<0.0001
**High-performance liquid chromatography analysis data (3)**						
Plasma MDA (mM)	4.4 ± 2.4	5.0 ± 2.5	0.009	4.3 ± 2.2	4.8 ± 2.0	0.083
Erythrocyte MDA (mM)	6.1 ± 6.2	6.3 ± 6.9	0.601	8.8 ± 8.6	9.4 ± 7.7	0.564
Urine MDA (mM)	2.9 ± 1.8	2.7 ± 1.5	0.380	2.7 ± 1.7	2.5 ± 1.3	0.207

Values are mean ± SD or number (percentage). Differences between the groups were compared using Student’s *t*-test for continuous variables and Chi-square tests for categorical variables. AP, alkaline phosphatase; BMI, body mass index; BUN, blood urea nitrogen; CRP, C-reactive protein; GOT, glutamate oxaloacetate transaminase; GPT, glutamate pyruvate transaminase; GT, γ-Glutamyl transferase; LDL-C, low-density lipoprotein cholesterol; ESR, erythrocyte sedimentation rate; MCH, mean corpuscular hemoglobin; MCHC, mean corpuscular hemoglobin concentration; MCV, mean corpuscular volume; MDA, malondialdehyde; PDW, platelet distribution width; RDW, red blood cell distribution width; RFS, recommended food score; TC, total cholesterol.

## Data Availability

Data are included in the article.

## Supplementary Materials

The following are available online at https://www.mdpi.com/article/10.3390/antiox10071132/s1, Figure S1, Classification of oxidative stress-related disease; Figure S2, Analysis of missing data; and Table S1, Comparison of healthy controls in the training set and those in the hold-out set.
