# Supplementary material for: A Machine Learning Algorithm for Quantitatively Diagnosing Oxidative Stress Risks in Healthy Adult Individuals Based on Health Space Methodology: A Proof-of-Concept Study Using Korean Cross-Sectional Cohort Data
Source: Antioxidants (Basel). 2021 Jul 16;10(7):1132. doi: 10.3390/antiox10071132 (PMC8301183; doi:10.3390/antiox10071132)
Supplement: Supplementary file 1 [file antioxidants-10-01132-s001.zip › antioxidants-1255281-supplementary.pdf]

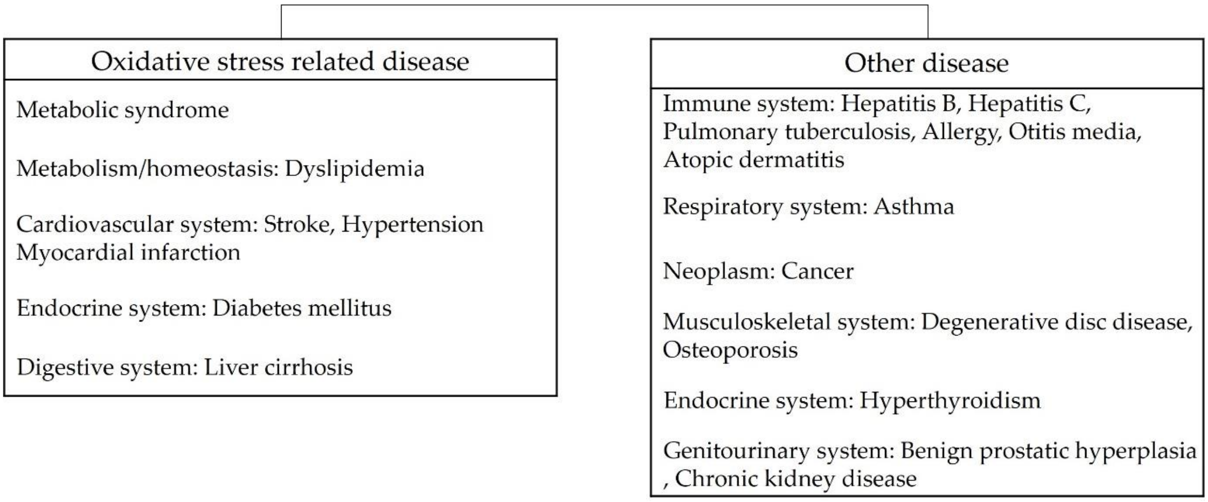

Figure S1. Classification of oxidative stress-related disease

(A)

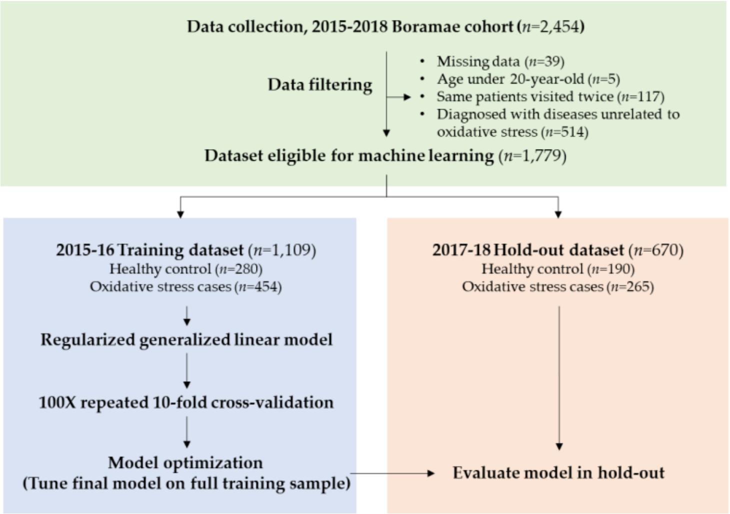

(B)

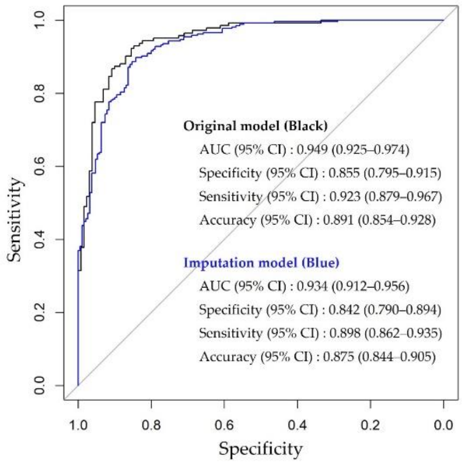

Selected features:

Age, Plasma MDA, BMI, RFS, HbA1c, GPT, GGT, Bilirubin, Albumin, WBC, RBC, Hb, RDW, Monocytes, Basophils, MCHC

(C)

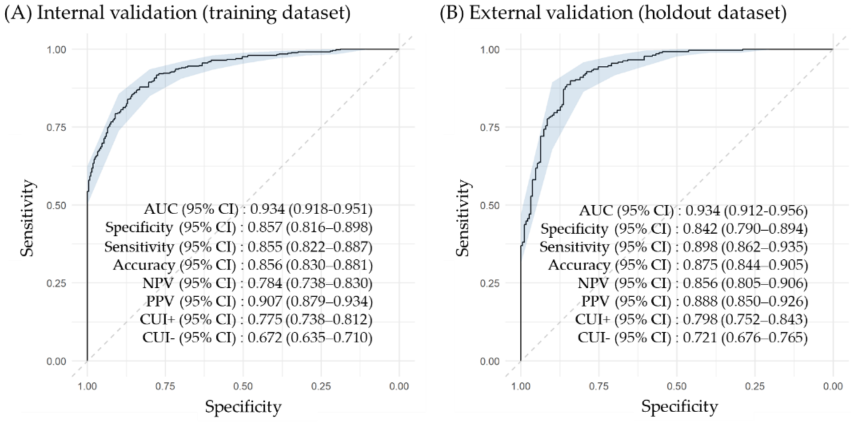

Figure S2. Analysis of missing data. (A) the schematic of the analysis, (B) selected features and performance test using receiver operating characteristic curve of best performing model, compared with the original model (p=0.376), and (C) internal and external validation. The diagonal line represents the reference line of 0.5. AUC, area under the curve; CI, confidence interval.

Table S1. Comparison of healthy controls in the training set and those in the hold-out set.

Features	Normal range	Healthy controls 2015–2016 Training set (*n* = 248)	Healthy controls 2017–2018 Hold-out set (*n* = 131)	*p*-value
**General characteristics (10)**				
Age (years)	-	42.7 ± 10.4	38.3 ± 10.0	0.0001
Sex (males; n, %)	-	101 (40.7)	47 (35.9)	0.358
Current smoker *n* (%)	-	23 (9.3)	5 (3.8)	0.053
Smoking duration (years)	-	4.5 ± 9.0	3.1 ± 6.8	0.091
Smoking pack-year	-	3.3 ± 7.6	2.4 ± 6.4	0.215
Current drinker *n* (%)	-	145 (58.5)	78 (59.5)	0.840
Recommended food score	-	21.6 ± 8.3	20.5 ± 8.7	0.215
Physical activity (min/day)	-	128.5 ± 120.1	119.2 ± 113.8	0.465
Body mass index (kg/m^2)		21.5 ± 2.2	21.2 ± 2.2	0.261
Body fat (%)		25.5 ± 6.4	26.0 ± 6.4	0.417
**Biochemical characteristics (14)**				
Albumin (g/dL)	3.5-5.2	4.3 ± 0.2	4.4 ± 0.2	0.001
Alkaline phosphatase (IU/L)	44-147	62.5 ± 17.8	62.3 ± 17.2	0.908
Bilirubin (mg/dL)	0.1-1.2	1.2 ± 0.5	1.1 ± 0.4	0.101
Blood urea nitrogen (mg/dL)	8-23	12.8 ± 3.5	11.6 ± 2.8	0.0004
Creatinine (μmol/L)	Male: 61.9-114.9<br>Female: 53-97.2	68.2 ± 14.1	66.1 ± 12.6	0.154
C-reactive protein (mg/dL)	0-3	0.1 ± 0.2	0.1 ± 0.3	0.787
γ-Glutamyl transferase (IU/L)	0-30	18.4 ± 15.0	17.7 ± 13.7	0.662
GOT (IU/L)	Male 0-40<br>Female 0-32	22.6 ± 6.8	26.7 ± 56.0	0.405
GPT (IU/L)	Male 0-41<br>Female 0-33	18.9 ± 12.0	18.6 ± 12.3	0.810
Glycosylated hemoglobin (%)	0-5.7	5.4 ± 0.3	5.3 ± 0.3	0.008
LDL-C (mmol/L)	0-3.5	3.0 ± 0.7	3.0 ± 0.7	0.971
Total cholesterol (mmol/L)	0-5.18	5.0 ± 0.8	5.0 ± 0.8	0.457
Total protein (g/dL)	6.4-8.3	7.1 ± 0.4	7.2 ± 0.3	0.002
Uric acid (mg/dL)	Male: 2.4-6.0<br>Female: 3.4-7.0	4.8 ± 1.2	4.7 ± 1.1	0.691
**Complete blood count data (16)**				
Basophils (%)	0.0-2.0	0.4 ± 0.3	0.5 ± 0.3	0.154
Eosinophils (%)	1.0-6.0	2.6 ± 2.2	2.7 ± 2.0	0.800
ESR (mm/h)	Male: 0-22<br>Female: 0-29	9.0 ± 8.2	9.4 ± 11.9	0.731
Hematocrit (%)	Male: 39-52<br>Female: 36-48	41.4 ± 4.2	42.7 ± 3.7	0.003
Hemoglobin (g/dL)	Male: 13-17<br>Female: 12-16	13.9 ± 1.6	14.2 ± 1.5	0.099
Lymphocytes (%)	17.0-46.0	35.3 ± 8.8	36.4 ± 7.9	0.224
MCH (pg)	Male: 27-33<br>Female: 26-32	30.3 ± 1.9	30.0 ± 1.9	0.208
MCHC (g/dL)	30.0-35.0	33.5 ± 1.0	33.2 ± 0.9	0.001
MCV (fL)	Male: 81-96<br>Female: 79-95	90.4 ± 4.4	90.6 ± 4.7	0.647
Monocytes (%)	2.0-8.0	5.7 ± 1.5	5.8 ± 1.4	0.736
Neutrophils (%)	38.0-78.0	56.0 ± 9.7	54.7 ± 8.4	0.201

Platelets (×10³/μL)	130-400	249.1	±	51.9	258.5	±	54.0	0.101
Platelet distribution width (%)	8.3-56.6	11.8	±	1.6	11.8	±	1.5	0.734
Red blood cell count (×10⁶/μL)	Male: 4.2-6.3<br>Female: 4.0-5.4	4.6	±	0.4	4.7	±	0.4	0.004
RDW (%)	Male: 12.2-16.1<br>Female: 11.8-14.5	13.1	±	1.0	13.1	±	1.1	0.998
White blood cell count (×103/μL)	4.0-10.0	5.2	±	1.3	5.2	±	1.3	0.959
**High-performance liquid chromatography analysis data (3)**								
Plasma MDA (mM)	-	4.4	±	2.4	4.3	±	2.2	0.622
Erythrocyte MDA (mM)	-	6.1	±	6.2	8.8	±	8.6	0.002
Urine MDA (mM)	-	2.9	±	1.8	2.7	±	1.7	0.369

Values are mean ± SD or number (percentage). Differences between the groups were compared using Student's *t*-test for continuous variables and Chi-square tests for categorical variables. AP, alkaline phosphatase; BMI, body mass index; BUN, blood urea nitrogen; CRP, C-reactive protein; GOT, glutamate oxaloacetate transaminase; GPT, glutamate pyruvate transaminase; GT, γ-Glutamyl transferase; LDL-C, low-density lipoprotein cholesterol; ESR, erythrocyte sedimentation rate; MCH, mean corpuscular hemoglobin; MCHC, mean corpuscular hemoglobin concentration; MCV, mean corpuscular volume; MDA, malondialdehyde; PDW, platelet distribution width; RDW, red blood cell distribution width; RFS, recommended food score; TC, total cholesterol.
